# Telemedicine (mobile-Health) as an alternative in the follow-up of patients in a randomized clinical trial for the treatment of cutaneous leishmaniasis in the state of Amazonas, Brazil^☆^

**DOI:** 10.1016/j.abd.2024.08.003

**Published:** 2025-01-09

**Authors:** Silmara Navarro Pennini, Jorge Augusto de Oliveira Guerra, Paula Frassinetti Bessa Rebello, Marília Rosa Abtibol-Bernardino, Amanda Canto Duarte, Higor Marques de Queiroz, Anette Chrusciak-Talhari, Maria das Graças Vale Barbosa Guerra, Sinésio Talhari

**Affiliations:** a Postgraduate Program in Tropical Medicine, Universidade do Estado do Amazonas, Manaus, AM, Brazil; b Fundação de Medicina Tropical Dr Heitor Vieira Dourado, Manaus, AM, Brazil; c Department of Teaching and Research, Fundação Hospitalar de Dermatologia Tropical e Venereologia Alfredo da Matta, Manaus, AM, Brazil; d Medicine Course Coordination, Faculdade Metropolitana de Manaus, Manaus, AM, Brazil; e Department of Maternal and Child Health, Faculty of Medicine, Universidade Federal do Amazonas, Manaus, AM, Brazil; f Medicine Course Coordination, Faculty of Medicine, Universidade Nilton Lins, Manaus, AM, Brazil; g Medicine Course Coordination, Escola Superior de Saúde, Universidade do Estado do Amazonas, Manaus, AM, Brazil

Dear Editor,

The technology and popularity of mobile phones have made them a useful tool for telemedicine, which is the practice of medicine carried out remotely using information technology, including the WhatsApp application, which is free, easy to use and has become one of the most widely used worldwide.^1^, ^2^

Mobile-Health (m-Health), has been used in some medical specialties, especially in Dermatology, where inspection is essential. Images of lesions recorded in photographs may be sufficient for the diagnosis, especially if corroborated by audio or text interaction with clinical information.^1^

During the COVID-19 pandemic, doctors, health units, and patients were not prepared to use telemedicine, but WhatsApp was seen as a possible interface and began to be used more frequently for this purpose. 1, 2

In October 2020, a clinical trial with new therapeutic options for cutaneous leishmaniasis (CL) was started by the authors, which coincided with the advance of the COVID-19 pandemic, making it difficult to follow up patients who would be assigned three evaluations (30, 90, and 180 days after starting treatment). Alternatively, WhatsApp was used to asynchronously exchange messages and send images of the lesions. Those who did not attend the appointments were contacted to reinforce the need to return for clinical and laboratory evaluation and to reschedule the appointment. At that time, they were asked to send photographs of the lesions and, if they did not attend the appointment, the images received were considered for clinical evaluation and the follow-up was then considered virtual.^3^ The objective of this publication is to describe the usefulness of the m-Health strategy in reducing follow-up losses in the aforementioned clinical trial.

A total of 49 patients were included in the clinical trial, of which 35 (71.4%) were evaluated through m-Health at some point during the follow-up, 18 of them in the experimental group and 17 in the control group. According to the time of the evaluation, an increasing number of consultations were performed through m-Health, 10 on D30, 19 on D90 and 24 on D180. Regarding the 24 patients whose last evaluation was virtual, 21 had an already healed lesion in the previous consultation, on D90, two were in the healing process and one showed therapeutic failure at that time. Those who did not return or were absent (Table 1) were considered as not attending in person or responding to the message via cell phone. The images sent by the patients were considered adequate and sufficient to assess the healing process (Fig. 1). Four patients did not answer telephone calls for the last visit (D180) and were visited at home. Two of these patients were at home and were evaluated; one answered a phone call made subsequently and the other had changed address and cell phone number and was considered lost to follow-up.

**Table 1:** Distribution of patients according to the type of return to follow-up consultation 30, 90 and 180 days after starting treatment, in a clinical trial for cutaneous leishmaniasis.

Type of return consultation	D30	D90	D180
In-person	30 (61.2%)	21 (42.8%)	22 (45.0%)
Virtual	10 (20.4%)	19 (38.8%)	24 (49.0%)
Home visit	‒	‒	2 (4.0%)
No return consultation	9 (18.4%)	9 (18.4%)	1 (2.0%)

**Fig. 1:**
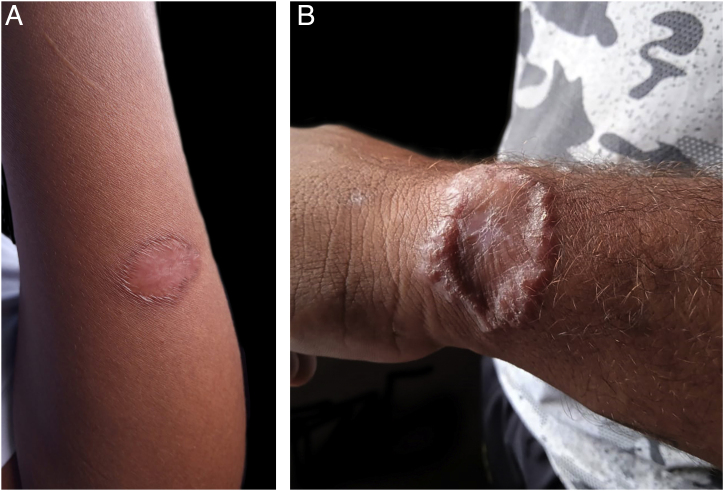
Photographs sent by patients: healed (A) and treatment failure (B).

A study on the implementation of teledermatology with 20,912 in-person and 4,512 remote consultations concluded that teledermatology seems to be an efficient tool for resolving dermatological problems, with a fast, effective and high-quality response to skin disease care.^4^

Cutaneous leishmaniasis is a neglected tropical disease. In the Amazon region, the CL transmission cycle occurs in the forest, affecting, for the most part, people who use extractivism for subsistence and who, due to geographical conditions and the long distances to be traveled, have difficulty both in carrying out treatment and remaining in follow-up in clinical trials, which ideally require 180 days of follow-up.^5^, ^6^ A clinical trial carried out in the region, without the use of telemedicine and outside the context of a pandemic, showed a rate of 7% patients lost to follow-up.^7^

A study conducted in rural areas of Colombia found that the implementation of an m-Health strategy for CL significantly increased the proportion of patients under surveillance from 4.2% (standard care) to 82.5%, as well as the proportion of patients with reports of adherence to treatment, adverse reactions to medications and therapeutic response.^8^

The use of the WhatsApp application in the asynchronous model or store-and-forward system (SAF) of telemedicine, where information or images are stored and evaluated later, at another time and place, proved to be a perfectly viable tool for use in the region, mainly because it does not depend on internet speed. The conditions in most rural areas of Amazonas do not allow access to synchronous consultations (video calls), in which the doctor-patient interaction occurs in real time, and also because in agriculture activities of most patients hamper this possibility.

The impact of the COVID-19 pandemic on research was demonstrated by Lasch et al. who demonstrated the reduction in the number of phase II and phase III clinical trials initiated in Europe and the United States as a whole and in clinical trials unrelated to COVID-19.^9^

The quality of the images and patient adherence are important for the method success. A good response to telephone contact was observed and, despite the lack of recommendations for photograph technique, the images sent made it possible to assess the clinical aspects of the lesions and were useful for decision-making. In some cases, it was only necessary to zoom in on the image. Under ideal conditions, patients can be instructed to capture images closer to the desired standard.

It is concluded that the m-Health strategy with the WhatsApp application made the CL clinical trial viable in a remote area during a pandemic situation, since it allowed rescuing 24 of the 27 patients who had been absent at the last consultation. Therefore, the prospect of using this strategy in future clinical research projects in hard-to-reach areas is foreseen, which may be an incentive for conducting more studies in these regions.

The study is part of a larger project, approved by the Research Ethics Committee CEP-FUHAM: CAAE 36533620.3.1001.0002; Opinion number. 4,663,792.

## Financial support

None declared.

## Acknowledgments

We thank the Fundação de Amparo à Pesquisa do Estado do Amazonas (FAPEAM) for a scientific initiation scholarship in technical cooperation with the Fundação Hospitalar de Dermatologia Tropical e Venereologia Alfredo da Matta (FUHAM).

## Authors' contributions

Silmara Navarro Pennini: Design and planning of the study; collection, analysis and interpretation of data; intellectual participation in the propaedeutic and/or therapeutic conduct of the studied cases; drafting and editing of the manuscript; approval of the final version of the manuscript.

Jorge Augusto de Oliveira Guerra: Intellectual participation in the propaedeutic and/or therapeutic conduct of the studied cases; drafting and editing of the manuscript; approval of the final version of the manuscript.

Paula Frassinetti Bessa Rebello: Critical review of the literature; intellectual participation in the propaedeutic and/or therapeutic conduct of the studied cases; drafting and editing of the manuscript.

Marília Rosa Abtibol-Bernardino: Collection, analysis and interpretation of data.

Amanda Canto Duarte: Critical review of the literature; collection and analysis of data.

Higor Marques de Queiroz: Critical review of the literature; critical review of the manuscript.

Anette Chrusciak-Talhari: Intellectual participation in the propaedeutic and/or therapeutic conduct of the studied cases.

Maria das Graças Vale Barbosa Guerra: Drafting and editing of the manuscript; critical review of the manuscript.

Sinésio Talhari: Research supervision; drafting and editing of the manuscript; critical review of the manuscript; approval of the final version of the manuscript.

## Conflicts of interest

None declared.
